# Static compliance of the respiratory system in COVID-19 related ARDS: an international multicenter study

**DOI:** 10.1186/s13054-020-03433-0

**Published:** 2021-02-08

**Authors:** Benoit Vandenbunder, Stephan Ehrmann, Michael Piagnerelli, Bertrand Sauneuf, Nicolas Serck, Thibaud Soumagne, Julien Textoris, Christophe Vinsonneau, Nadia Aissaoui, Gauthier Blonz, Giuseppe Carbutti, Romain Courcelle, Alain D’hondt, Stephane Gaudry, Julien Higny, Geoffroy Horlait, Sami Hraiech, Laurent Lefebvre, Francois Lejeune, Andre Ly, Jean-Baptiste Lascarrou, David Grimaldi, for the COVADIS study group, Benoit Vandenbunder, Stephan Ehrmann, Michael Piagnerelli, Bertrand Sauneuf, Nicolas Serck, Thibaud Soumagne, Julien Textoris, Christophe Vinsonneau, Nadia Aissaoui, Gauthier Blonz, Giuseppe Carbutti, Romain Courcelle, Alain D’hondt, Stephane Gaudry, Julien Higny, Geoffroy Horlait, Sami Hraiech, Laurent Lefebvre, Francois Lejeune, Andre Ly, Jean‑Baptiste Lascarrou, David Grimaldi, Patrick Biston, Gwenhael Colin, Oriane de Maere, Nathan Ebstein, Frederic Foret, Thibault Helbert, Jean-Baptiste Mesland, Celine Monard, Nicolas Mongardon, Gregoire Ottavy, Thomas Pasau, Gael Piton, Zoe Pletschette, Ester Ponzetto, Caroline Sejourne, Piotr Szychowiak, Xavier Souloy, Aude Sylvestre, Nicolas Tartrat, Cedric Vanbrussel

**Affiliations:** 1 Groupe des anesthésistes réanimateurs, Hôpital Privé d’Antony, Antony, France; 2 CHRU Tours, Médecine Intensive Réanimation, CIC INSERM 1415, CRICS-TriggerSEP research network, and INSERM, Centre d’étude des pathologies respiratoires, U1100, Université de Tours, Tours, France; 3 Intensive Care, CHU-Charleroi, Marie Curie, Université Libre de Bruxelles, 140, chaussée de Bruxelles, 6042 Charleroi, Belgium; 4 Réanimation - Médecine Intensive, Centre Hospitalier Public du Cotentin, BP208, 50102 Cherbourg-en-Cotentin, France; 5 Unité de soins intensifs, Clinique Saint Pierre, Ottignies, Belgium; 6 Médecine Intensive Réanimation, CHU Besançon, 3 Boulevard FLEMING, 25030 Besançon, France; 7 Service de réanimation, Hospices Civils de Lyon, 5 Place D’Arsonval, Lyon, France; 8 Laboratoire Commun de Recherche bioMérieux-Hospices Civils de Lyon, Université de Lyon 1, EA7426 PI3 Lyon, France; 9 Service de Médecine Intensive Réanimation Unité de Sevrage Ventilatoire Et Réhabilitation Centre Hospitalier de BETHUNE, 27 Rue Delbecque, 62660 Beuvry, France; 10 Médecine Intensive Réanimation, Hôpital Européen Georges Pompidou, Paris Centre U 970 PARCC, Paris, France; 11 Médecine Intensive Reanimation, District Hospital Center, Boulevard Stephane Moreau, 85000 La Roche Sur Yon, France; 12 Unité de Soins Intensifs, CHR Mons-Hainaut, Mons, Belgium; 13 Unité de Soins Intensifs, Centres Hospitaliers de Jolimont, La Louvière, Belgium; 14 Unité de Soins Intensifs, CHU Ambroise Paré, Mons, Belgium; 15 Réanimation médico-Chirurgicale CHU Avicennes, Université Sorbonne Paris Nord, Bobigny, France; 16 Unité de Soins Intensifs, CHU Dinant Godinne, Site Dinant, Dinant, Belgium; 17 Unité de Soins Intensifs, CHU Dinant Godinne, Site Godinne, Yvoir, Belgium; 18 Médecine Intensive Réanimation, Assistance Publique - Hôpitaux de Marseille, Hôpital Nord, 13015 Marseille, France; 19 Centre d’Etudes et de Recherches sur les Services de Santé et qualité de vie EA 3279, Aix-Faculté de médecine, Marseille Université, 13005 Marseille, France; 20 Réanimation Polyvalente Centre Hospitalier du Pays D’Aix, Aix en Provence, France; 21 Unité de Soins Intensifs, Clinique Notre Dame de Grâce, Gosselies, Belgium; 22 Service D’anesthésie-réanimation Chirurgicale, Unité de réanimation Chirurgicale Polyvalente, Hôpitaux Universitaires Henri Mondor, Créteil, France; 23 Médecine Intensive Réanimation, CHU Nantes, 30 Boulevard Jean Monnet, 44093 Nantes Cedex 9, France; 24 Soins Intensifs, Hôpital Erasme, ULB, Route de Lennik 808, 1070 Bruxelles, Belgium

**Keywords:** SARS-COV-2, Plateau pressure, Respiratory mechanics, Mechanical ventilation, PEEP, Mortality, ICU

## Abstract

**Background:**

Controversies exist on the nature of COVID-19 related acute respiratory distress syndrome (ARDS) in particular on the static compliance of the respiratory system (Crs). We aimed to analyze the association of Crs with outcome in COVID-19-associated ARDS, to ascertain its determinants and to describe its evolution at day-14.

**Methods:**

In this observational multicenter cohort of patients with moderate to severe Covid-19 ARDS, Crs was measured at day-1 and day-14. Association between Crs or Crs/ideal body weight (IBW) and breathing without assistance at day-28 was analyzed with multivariable logistic regression. Determinants were ascertained by multivariable linear regression. Day-14 Crs was compared to day-1 Crs with paired t-test in patients still under controlled mechanical ventilation.

**Results:**

The mean Crs in 372 patients was 37.6 ± 13 mL/cmH_2_O, similar to as in ARDS of other causes. Multivariate linear regression identified chronic hypertension, low PaO_2_/FiO_2_ ratio, low PEEP, and low tidal volume as associated with lower Crs/IBW. After adjustment on confounders, nor Crs [OR 1.0 (CI 95% 0.98–1.02)] neither Crs/IBW [OR 0.63 (CI 95% 0.13–3.1)] were associated with the chance of breathing without assistance at day-28 whereas plateau pressure was [OR 0.93 (CI 95% 0.88–0.99)]. In a subset of 108 patients, day-14 Crs decreased compared to day-1 Crs (31.2 ± 14.4 mL/cmH_2_O vs 37.8 ± 11.4 mL/cmH_2_O, *p* < 0.001). The decrease in Crs was not associated with day-28 outcome.

**Conclusion:**

In a large multicenter cohort of moderate to severe COVID-19 ARDS, mean Crs was decreased below 40 mL/cmH_2_O and was not associated with day-28 outcome. Crs decreased between day-1 and day-14 but the decrease was not associated with day-28 outcome.

## Introduction

Coronavirus disease 2019 (COVID-19) caused by the severe acute respiratory syndrome coronavirus 2 infection can have different clinical presentations but respiratory symptoms predominate, especially in patients admitted to intensive care units (ICU) [1]. The clinical presentation of the respiratory disease appeared at the beginning of the pandemic to be relatively homogenous: It involves mostly overweighed men aged 50 years or more, with cardiovascular comorbidities, and is characterized by severe hypoxemia and radiological ground glass opacities [2].

For the peculiar COVID-19 related acute respiratory distress syndrome (ARDS), some experts hypothesized that it could be separated in two main phenotypes according to lung mechanical properties: Patients at the early phase of the disease would have a high pulmonary compliance, whereas others patients may have low compliance, upfront or as transition from the first phenotype, because of self-induced lung injury [3]. Indeed, COVID-19 hypoxemia seemed not to be fully explained by loss of aerated lung volume [4]. The classical “baby-lung” concept has been challenged as well as the use of the ARDS terminology to describe COVID-19 hypoxemic pneumonia [5]. In line with those pathophysiological reasoning, experts exerted physician to tailor respiratory therapy [such as tidal volume (Vt), positive end expiratory pressure (PEEP) or prone positioning] to each adequate phenotype at an individual level [6].

However, in autopsy studies, the predominant pulmonary histologic pattern of COVID-19 appeared to be diffuse alveolar damage, a characteristic feature of ARDS whatever the cause, associated with a high frequency of arterial thrombi [7–9]. Those results are compatible with autopsies being performed predominantly at late stages of the disease.

The two phenotypes concept has also been challenged by clinical data from monocentric studies with a limited number of patients showing that the mean compliance of the respiratory system (Crs) in COVID-19 ARDS patients was decreased around 30–40 mL/cmH_2_O [10, 11]. There is then still intense debate about the actual Crs of COVID-19 patients’ and subsequent therapeutic implications [12–14].

The multicenter prospective COVADIS study [15] included patients suffering from moderate to severe COVID-19 ARDS. We hypothesized that high Crs would be associated with the likelihood of breathing without assistance at day-28.

Our primary aim was to compare patients’ characteristics and outcome according to low or high Crs. Secondary aims were to analyze the determinants of day-1 Crs in COVID-19 patients with moderate to severe ARDS and to describe the evolution of compliance at day-14 in a subset of patients.

## Patients and methods

This study was compliant with STROBE guidelines.

### Study design

This multicentric prospective observational study was performed in 21 ICUs in France (*n* = 12) and Belgium (*n* = 9). The COVID-19 pandemic began in France in the second week of March 2020 and one week later in Belgium.

### Patient population

Inclusion criteria were:

- Age older than 18 years,
- moderate to severe ARDS according to Berlin definition [16] (PaO_2_/FiO_2_ ratio < 200 mmHg with a PEEP of at least 5 cmH_2_O receiving invasive ventilation),
- positive SARS-CoV-2 RT-PCR.

Non-inclusion criteria were:

- Cardiac arrest before ICU admission,
- Extra corporeal membrane oxygenation (ECMO) requirement within the first 24 h of ICU admission,
- Chronic obstructive pulmonary disease with gold class 3 or 4 [17], or home oxygen therapy.

### Data collection

The collected data have been described elsewhere [15]. Briefly, patients were included in participating ICUs between March 10, 2020 and April 15, 2020. We recorded demographics data and comorbidities using the Charlson comorbidity index [18]. We collected duration since symptoms onset and presence of coinfection. We recorded after optimization the following mechanical ventilation (MV) settings after intubation in supine position and initial ventilation optimization: total PEEP, plateau pressure (Pplat), Vt. We recorded administration of advanced therapies for acute respiratory failure during the ICU stay (neuromuscular blocking agents, inhaled pulmonary vasodilators, prone-positioning, and ECMO).

We calculated from measured variables the driving pressure as DP = Pplat-PEEP and the compliance of the respiratory system as Crs = Vt/DP in mL/cmH_2_O. To take into account the height of the patients, we calculated also the Crs/ideal body weight (IBW).

In patients still on volume/pressure-assisted controlled MV at day-14, we measured and calculated the same variables.

General guidelines for ARDS management were followed in all centers: targeting a Vt of 6 mL/kg of ideal body weight (IBW), limited plateau pressure, prone positioning for severe hypoxemia [19]. NMBA were used with slight differences across centers [20]. The setting of PEEP was not protocolized and was left at the discretion of the attending physician.

### Primary objective and outcome

The primary objective was to assess the outcome of COVID 19 patients requiring invasive mechanical ventilation according to initial Crs.

The prespecified primary endpoint was the number of ventilator free days (VFD) at day-28 [21] dichotomized in breathing without assistance (VFD ≥ 1) or not (VFD = 0).

### Secondary outcomes

- Day-14 MV mode according to the following 4 pre-defined categories: (1) spontaneous breathing while extubated, (2) pressure support ventilation, (3) patient under volume/pressure-assisted controlled MV or ECMO, and (4) death
- Day-14 survival
- Day-28 survival
- Need for ECMO
- Pulmonary embolism

### Statistical analysis

Discrete data were described by counts and percentage and compared using the Chi-square or Fisher’s exact tests, as appropriate. Continuous data were described by the mean and standard deviation or by the median and interquartile range (IQR) and compared by the *t *test or by the Mann–Whitney test as appropriate.

To identify the determinants of day-1 Crs, we compared patients with the lowest Crs to patients with the highest. We further performed a multivariate linear regression including in the model variables describing patients’ characteristics and ventilatory setting (Vt and PEEP), the Crs being the dependent continuous variable. We did the same analysis, the Crs/IBW being the dependent variable. We performed a backward selection eliminating variables with a *P* value above 0.10. Pplat and DP were not included in this analysis as mathematically linked to Crs and not set by the physician. Visual inspection of residues distribution was used to ensure the quality of the regressions.

We split Crs into quintiles to test its trend for association with day-28 outcome. Finally, we performed a multivariate backward logistic regression to analyze the association between breathing without assistance at day-28 and Crs (as a continuous variable). We included in the models variables associated with the primary endpoint in univariate analysis with a *P* value < 0.10 and we forced Crs as a continuous variable into the model, we did the same replacing Crs by Crs/IBW. Homesher−Lemeshow test and visual inspection were used to ensure the quality of the regressions. Backward selection was performed as described above. We included Pplat and Crs in the same model despite collinearity following published method [22, 23] assuming that if one of these two variables remained significantly correlated with the primary endpoint, this variable would be more informative than the other.

After reviewing of the manuscript, we performed post hoc analyses. We analyzed the association between Crs and our primary endpoint in the subgroups of patients with severe and moderate ARDS (*P*/*F* ≤ 100 mmHg and *P*/*F* between 101 and 200 mmHg). We also analyzed the correlation between *P*/*F* ratio and Crs across three categories of PEEP (5–8, 9–12 and above 12 cmH_2_O) using Pearson correlation.

To analyze the evolution of Crs at day-14, we measured day-14 Crs in patients under controlled MV as described above. We compared paired respiratory data (Vt, PEEP, DP, Crs) using the paired t-test or Wilcoxon ranking test according to distribution. We analyzed the association between day-1 Crs and day-14 Crs by univariate linear regression and by comparing delta Crs according to final outcome (breathing without assistance, still under invasive ventilation or death).

No imputation strategy was used for missing data. A *P* value < 0.05 was considered significant.

All analyses were performed using Stata (version 16, StataCorp, College Station, TX, USA).

### Ethics statement

This study was approved by appropriate regulatory committee in France and Belgium in accordance with national regulation (2217488 and P2020/253). Each patient was informed about the study. In case of incompetency, next of kin were informed. The requirement for written informed consent was waived.

### Role of the funding source

This study was not funded by any sources.

## Results

**Fig. 1:**
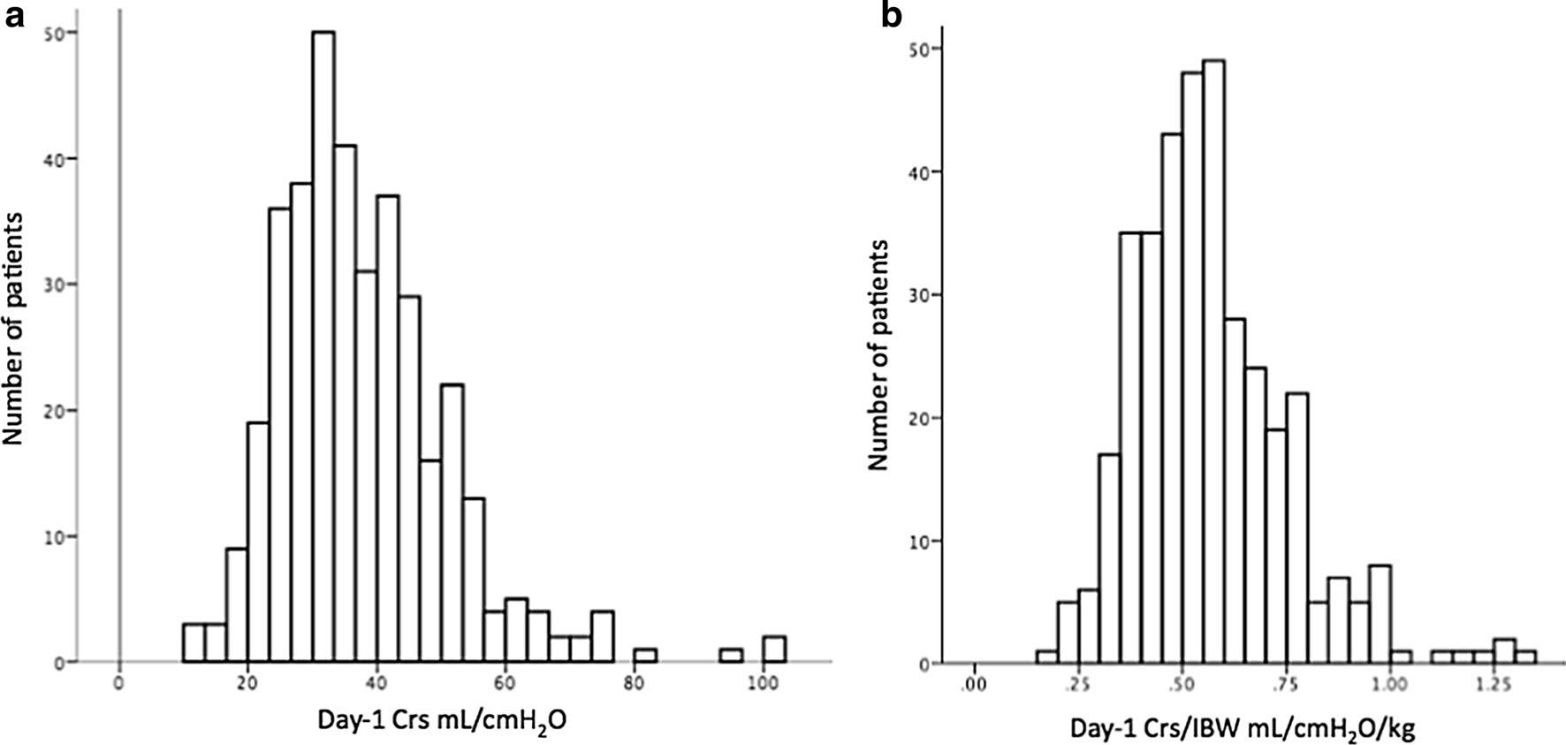
Distribution of day-1 Crs (**a**) and day-1 Crs/IBW (**b**). Crs: compliance of the respiratory system, IBW: ideal body weight

Among the 416 patients included in the study, one withdrew consent and we could calculate the Crs in 372 (Additional file 1). The mean value of Crs was 37.6 (± 13) mL/cmH_2_O, with a unimodal distribution (Fig. 1).

### Baseline characteristics according to compliance (Table 1 and Additional file 1: Table 1)

**Table 1:** Patients’ characteristics according to day-1 compliance of the respiratory system

*n* = 372	Crs < 35.4 *n* = 186	Crs > 35.4 *n* = 186	*P* value^a^
Age, mean ± SD	63.5 ± 10	63.2 ± 10.8	0.78
Gender, men, *n* (%)	130 (70)	154 (83)	0.005
Body mass index, kg/m^2^, mean ± SD	29.9 ± 5.1	29.5 ± 5	0.47
Hypertension, *n* (%)	116 (62)	94 (51)	0.02
Pulmonary chronic disease, *n* (%)	24 (13)	26 (14)	0.88
Charlson comorbidity index, median (IQR)	1 (0–2)	1 (0–2)	0.18
Charlson comorbidity index			0.11
0	69 (37)	86 (46)	
1	53 (29)	38 (20)	
≥ 2	64 (34	62 (33)	
Time from symptoms onset, days, median (IQR)	7 (5–10)	8 (5–10)	0.02
Coinfection, *n* (%)	19 (10)	23 (12)	0.62
PaO_2_/FiO_2_ (mmHg), mean ± SD	123 ± 48	132 ± 53	0.07
Tidal volume (ml/kg IBW), mean ± SD *n* = 180/185	6.2 ± 0.9	6.3 ± 0.8	0.12
PEEP (cmH_2_O), mean ± SD	11.1 ± 2.9	12 ± 2.6	0.002
Plateau pressure (cmH_2_O), median (IQR)	26 (23–28)	22 (20–24)	< 0.001
Driving Pressure (cmH_2_O), median (IQR)	14 (12–16)	9.5 (8–11)	< 0.001
Inhaled nitric oxide, *n* (%)	21 (11)	23 (12)	0.87
Prone positioning, *n* (%)	152 (82)	147 (79)	0.51
Neuromuscular blocking agents, *n* (%)	162 (87)	151 (81)	0.16
Antiviral treatment, *n* (%)	143 (77)	158 (85)	0.06
Corticosteroids^b^, *n* (%) *n* = 175/182	34 (19)	43 (24)	0.37
Macrolides	112 (60)	116 (62)	0.75

*SD* standard deviation, *IQR* interquartile range, *IBW* ideal body weight; *Crs *static compliance of the respiratory system

^a^*P *value was calculated by Fisher exact test, *t *test or Mann–Whitney test as appropriate

^b^Some patients were included in a RCT steroids versus placebo, steroids were mostly given late in the ICU stay

We compared patients’ characteristics according to Crs dichotomized on the median value (35.4 mL/cmH_2_O). As shown in Table 1, compared to the patients with highest Crs, patients with the lowest Crs were more frequently women, suffered more frequently from chronic hypertension and had a slightly lower PaO_2_/FiO_2_ ratio, a lower PEEP with a higher Pplat and accordingly a higher DP. Patients were similarly treated with low Vt, large use of prone positioning and neuromuscular blocking agents.

We then analyzed the determinants of compliance (kept as a continuous variable) through multivariate linear regression and observed that female sex and chronic hypertension were associated with lower compliance whereas higher PEEP and Vt were associated with a higher compliance. Of note, neither BMI, pulmonary chronic disease nor duration of symptoms were associated with Crs (Additional file 2). To take into account the size of the patients, we analyzed also the determinants of the compliance/IBW ratio. In this analysis, sex was not associated with Crs/IBW whereas *P*/*F* ratio was (Additional file 2).

Finally, as Crs is modified by PEEP, we looked at the correlation between Crs/IBW and *P*/*F* according to three levels of PEEP. We observed that only the patients with a low PEEP (5–8 cmH_2_O) had a significant correlation between Crs/IBW and *P*/*F* ratio (Additional file 3).

### Outcome according to compliance (Table 2, Table 3, Fig. 2)

**Table 2:** Outcome according to day-1 compliance of the respiratory system

	Crs < 35.4 *n* = 186	Crs > 35.4 *n* = 186	*P* value^a^
Breathing without assistance at day-28, *n* (%)	66 (36)	81 (44)	0.17
Day-28 VFD, median (IQR)	0 (0–11)	0 (0–12.5)	0.20
Day-14 Ventilatory mode			
Death	50 (27)	38 (20)	0.48
Controlled or VV-ECMO	60 (33)	65 (35)	
Pressure support	38 (21)	42 (23)	
Extubated	35 (19)	41 (22)	
Alive at day-14, *n* (%)	135 (73)	148 (80)	0.14
Alive at day-28, *n* (%)	110 (60)	131 (71)	0.03
Pulmonary embolism, *n* (%)	18 (10)	33 (18)	0.03
VV-ECMO, *n* (%)	31 (17)	15 (8)	0.02

*IQR* interquartile range, *VV-ECMO* veno-venous extracorporeal membrane oxygenation, *VFD* ventilator free days, *Crs* static compliance of the respiratory system

^a^*P *value was calculated by Fisher exact test, *t *test or Mann–Whitney test as appropriate

**Table 3:** Factors associated with breathing without assistance at day-28

	Adjusted OR^a^	CI 95%^a^
Age, per year	0.95	0.93–0.97
Gender, men	0.5	0.3–0.8
PaO_2_/FiO_2_ per mmHg	1.006	1.002–1.01
Plateau pressure per cmH_2_O	0.93	0.88–0.99
Model using absolute values of Crs		
Crs per mL/cmH_2_O	1.0	0.98–1.02
Model using indexed values of Crs/IBW		
Crs/IBW per mL/cmH_2_O/kg IBW	0.63	0.13–3.1

*OR* odds ratio, *CI* confidence interval, *Crs* static compliance of the respiratory system

^a^Adjusted odds ratios (ORs), and their confidence interval (CI) were determined using multivariate logistic regression with backward selection. Variables entered in the models were age, sex, chronic hypertension, Charlson comorbidity index, plateau pressure, compliance of the respiratory system, and PaO_2_/FiO_2_ ratio in model 1 replacing Crs by Crs/IBW in model 2. As Crs/IBW was not retained in the final model, OR are identical for the other variables

*n*  = 365 and 359 patients for model 1 and 2, respectively

**Fig. 2:**
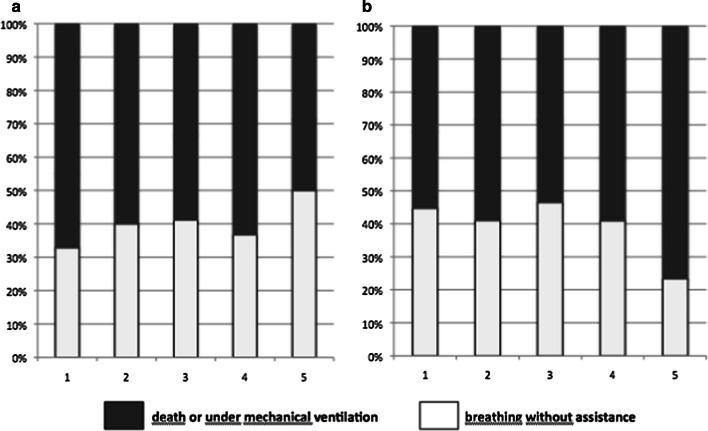
Proportion of patients breathing without assistance at day-28 according to Crs (**a**, *p* for trend = 0.11) and Pplat (**b**, *p* for trend = 0.03) quintiles

The proportion of patients breathing without assistance, as well as the day-28 VFD, were similar between the two groups (Table 2); however, more patients with low Crs had died at day-28.

Among several secondary outcomes, we observed that patients with the lowest Crs required more often ECMO during ICU stay but overall, the mechanical ventilation mode at day 14 was not different. Pulmonary embolism was more frequently diagnosed in patients with the highest compliance.

Divided into quintiles, Crs was not associated with breathing without assistance at day-28 whereas Pplat was (Fig. 2). To adjust for confounders, we performed a multivariate logistic regression of variables associated with breathing without assistance at day 28 including age, sex, chronic hypertension, Charlson comorbidity index, PaO_2_/FiO_2_ ratio, Pplat, and Crs (Table 3). This analysis showed that Crs was not associated with likelihood of breathing without assistance at day-28, [OR 1.0 (CI 95% 0.98–1.02)] whereas plateau pressure was negatively associated with [OR 0.93 (CI 95% 0.88–0.99)]. Sensitivity analysis considering Crs as a dichotomized variable provided the same results (data not shown). In a post hoc multivariate analysis including the same co-variables, Crs was not independently associated with Day-28 survival [OR 1.01 (CI 95% 0.98–1.03)] whereas age, Pplat, PaO_2_/FiO_2_ ratio, and Charlson Comorbidity index were. Finally, in the subgroup of severe ARDS Crs was similar in patients breathing without assistance at day-28 and in patients who did not (38.7 ± 11.3 vs 35.1 ± 11.8 mL/cmH_2_O, *P* = 0.11), the difference was even less pronounced in moderate ARDS patients (data not shown).

### Day 1-day 14 evolution of compliance (Table 4, Fig. 3)

**Table 4:** Change in respiratory system mechanics from day-1 to day-14

*n* = 108	Day 1	Day 14	*P *value^a^
Positive end expiratory pressure (cmH_2_O), mean ± SD	11.8 ± 2.7	10.3 ± 2.8	< 0.001
Plateau pressure (cmH_2_O), median (IQR)	23.5 (21–27)	23.5 (20–26.5)	0.49
Driving pressure (cmH_2_O), median (IQR)	11 (9–14)	13 (10–16)	< 0.001
Tidal volume (ml/kg IBW), mean ± SD	6.2 ± 0.8	5.6 ± 1.5	< 0.001
Crs (mL/cmH_2_O), mean	37.8 ± 11.4	31.2 ± 14.4	< 0.001

*SD* standard deviation, *IQR* interquartile range, *IBW* ideal body weight, *Crs* static compliance of the respiratory system

^a^*P *value was calculated by paired t-test or Wilcoxon test as appropriate

**Fig. 3:**
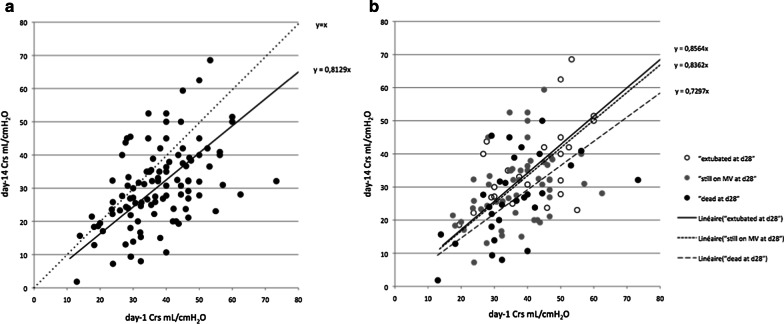
Relation between day-1 and day-14 Crs (*n * = 108). **a** shows the regression line (continuous) between day-14 and day-1 Crs compared to the *y* = *x* (dotted line). **b** shows the same data according to three day-28 outcomes: patients extubated (empty circles) patients still on mechanical ventilation (grey circles) and patients who died (black circles) and their respective regression lines

At day-14, Crs could be calculated in 108 patients still in controlled ventilation mode. The individual day-14 Crs was lower and strongly correlated with the day-1 Crs (*R*^2^ = 0.87 *p* < 0.001, Fig. 3a). Indeed, the mean Crs decreased from 37.8 ± 11.4 mL/cmH_2_O to 31.2 ± 14.4 mL/cmH_2_O (*p* < 0.001, paired *t *test). This decrease was explained by the increase in driving pressure as Vt decreased between day-1 and day-14. Conversely, Pplat was similar between day-1 and day-14 (Table 4).

The decrease of Crs between day-1 and day-14 was similar in patients that were extubated before day-28 and in those that were still under mechanical ventilation at this time point. Patients who died before day-28 had a slightly more pronounced decrease of Crs (Fig. 3b). This translated into a higher, although not significant, difference in Crs between day 14 and day 1: − 5.6 ± 12.2; − 5.8 ± 13.8; − 8.8 ± 12.1 mL/cmH_2_O (p = 0.55) in patients extubated, still on mechanical ventilation or deceased at day-28, respectively.

## Discussion

In this multicenter observational study of moderate to severe ARDS complicating COVID-19, our observations were: mean static compliance of the respiratory system was 37.6 mL/cmH_2_O with a monomodal distribution, while PEEP was set between 10 and 15 cmH_2_O for 78% of patients and Vt was tightly set between 6 and 7 mL/kg IBW. After adjustment, day-1 Crs was not associated with neither the chance of breathing without assistance at day-28 nor day-28 survival, whereas Pplat was. At day-14, in patients still in controlled ventilation mode, compliance had decreased in average but this decrease was not associated with day-28 status.

Patients’ characteristics in our cohort were similar to previous findings in other countries [24, 25]. Patients were mostly overweighed males between 50 and 70 years of age, with mostly mild cardiovascular comorbidities. In line with ARDS guidelines [19], physicians set Vt near 6 mL/kg of IBW, PEEP at moderate to high level, used largely prone positioning and paralysis. Thus, in this large, multicentric, international cohort of COVID-19 ARDS patients, one may consider typical, the mean compliance at day-1 after intubation was 37.6 mL/cmH_2_O. Of note this measure was done after ventilation optimization (Vt and PEEP setting) but not on a prespecified level of PEEP. It seems that for a given patient with COVID-19 ARDS, Crs is not that much influenced by PEEP even if in some individuals, a great variability has been observed when comparing 2 PEEP levels [26–28]. Early small monocentric studies reported mean Crs as high as 50 mL/cmH_2_O [4, 26, 29], but larger albeit still monocentric studies reported lower values ranging from 28 to 44 mL/cmH_2_O [10, 11, 30, 31]. In a large multicenter study including COVID-19 ARDS published at the date of writing, median compliance was 35 mL/cmH_2_O but could be measured in only 40% (*n* = 296) of the patients [32]. We found exactly the same median compliance (35.4 mL/cmH_2_O) in a larger number of patients with a high completion rate of data (nearly 90%). In a report of 300 ARDS patients from seven ICUs in Italy, median compliance was 41 mL/cmH_2_O, slightly higher than the one we observed [33]. As highlight by others, this mean compliance is similar [12, 32] or slightly higher [28, 33] to that observed in non-COVID-19 ARDS [34] contradicting the idea of a specifically high compliance in COVID-19 ARDS. We may hypothesize that at the beginning of the pandemic, early intubation was of common practice and led to an overestimation of the mean compliance of COVID-19 ARDS due to intubation of patients with low severity [35, 36]. Another hypothesis would be that the high initial rate of pulmonary embolism in COVID-19 [37, 38] before increase of thrombophrophylaxis [39] lowered the PaO_2_/FiO_2_ ratio and explained in some patients the discrepancy between severity of hypoxemia and alteration of respiratory mechanics.

We identified determinants of day-1 Crs. Interestingly, chronic hypertension was associated with a lower Crs but our study was not designed to provide explanation for these findings. Female sex was associated with a lower compliance due to the association between compliance and height. Indeed, the sex was not associated with the static compliance/IBW ratio. Vt and PEEP were associated with higher compliance (not meaning a causal relationship), but we may hypothesize that high PEEP was associated with significant alveolar recruitment although this phenomenon is not constant in COVID-19 ARDS [26, 30]. We observed a univariate association between compliance and *P*/*F* ratio, which was more pronounced in low level of PEEP, suggesting that some of these patients could have been recruitable. PEEP-induced recruitment may be evaluated by the recruitment-to-inflation ratio to more precisely analyze the relationship between PEEP setting and Crs; however, such detailed lung mechanics assessment was beyond the scope of this multicenter large-scale study [40].

Regarding Vt, the association with higher Crs is counterintuitive and probably reflects that the investigators decreased Vt to limit Pplat as a consequence of low Crs. Interestingly, we were able to confirm in a large cohort and after adjustment the lack of association between symptom duration and Crs [30].

Crs was not associated with the likelihood of breathing without assistance or with survival at day-28. This is in line with findings in classical ARDS as highlighted in the Berlin consensus paper where compliance did not add to the mortality prediction in severe ARDS [16]. Conversely, in our study, Pplat was strongly associated with day-28 outcomes (breathing without assistance and survival) even in multivariate analyses. It is known that high Pplat (above 28–30 cmH_2_O) is associated with ARDS mortality and thus guidelines recommend to target a Pplat below this threshold [19, 41], our study could, however, plead for a COVID-19-specific lower Pplat threshold as the association between Pplat and outcome was observed despite 90% of the present cohort having a Pplat ≤ 28 cmH_2_O. We observed a limited dispersion of the respiratory variables, which ensure a certain degree of homogeneity. However, it may preclude the generalizability of our findings in patients, which could have a strikingly different driving pressure as a result of higher Vt and/or lower PEEP. This has been already advocated as a possible explanation of discrepancy regarding the importance of DP as a prognostic factors in ARDS [23].

The last result is our original data on Crs evolution up to day 14. We observed in a subset of patients that Crs decreased between these two time points evoking either a fibrotic evolution of “late ARDS” or a loss of lung tissue aeration due to worsening lung disease [42, 43]. Few studies have provided repeated measurement of Crs: It seems not decrease at day 5 and 7 [10, 11]. In the multicenter study of Ferrando et al., a small subset of patients had Crs measurement until day 14 (*n* = 61) and Crs seemed to decrease after day 10 [32]. However, in these three studies, paired data were not shown, making difficult the interpretation of the results. Conversely, we analyzed paired values of compliance and observed a clear decrease. This observation is remarkable as, in the same time, physicians markedly decreased the Vt and the PEEP in order to keep Pplat in the same range that at day-1. This suggests that the decrease in Crs had been minimized by the prevention of end inspiratory overdistension. Maybe due to this adaptation and/or a lack of power, the decrease in Crs was not associated with outcome. Despite the lack of association with day-28 survival, the decrease in Crs could be associated with long-term respiratory sequelae and this should be analyzed in further studies. The main limit interpreting this set of results is that they concern less than 1/3 of the patients, the others being either dead, extubated, or on weaning process at day 14. This unavoidable bias limits the interpretation to a specific subset of patients, in whom, even at day-14, the data completion rate was as high as 85% (108/125) of day-14 Crs measurements.

Finally, we highlight the limitations of our observational study: The respiratory settings and patients’ management were not standardized although collected variables suggest high similarity in treatment strategies and adherence to ARDS guidelines. Non-measured confusion biases may exist anyway. We did not collect any ICU specific severity score but these scores have been developed to compare patients with different diseases in the ICU; furthermore, the Charlson Comorbidity index associated with gender and age has been shown to predict mortality with good accuracy and thus reflecting severity of disease [44]. Missing data, albeit scarce, may impact our results. With the choice of a pragmatic design, favoring feasibility during the COVID-19 crisis, we strongly limited the number of collected variables so that we were not able to report important but more complicated data such as transpulmonary pressure, recruitability, shunt fraction, or hemodynamic parameters as well as daily ventilator settings.

## Conclusion

In moderate to severe ARDS COVID-19 patients, we observed a unimodal distribution of the compliance of the respiratory system around a mean value of 37 mL/cmH_2_O as usually observed in non-COVID-19 ARDS. Higher compliance values were not associated with faster weaning of mechanical ventilation nor with improved survival in multivariate analyses. Mean compliance decreased from day-1 to day-14. Further studies are needed to analyze the consequence of such evolution.

## Supplementary Information

**Additional file 1**: Flow chart of the study.

**Additional file 2**: Determinants of day-1 Compliance and Compliance/IBW.

**Additional file 3**: Crs, Crs/IBW and *P*/*F* ratio according to Peep level.

## Data Availability

D. Grimaldi and JB. Lascarrou had full access to all the data in the study and had final responsibility for the decision to submit for publication. The database will be public within 3 months after publication at https://icucovadis.com.
