# Supplementary figures and images for: Static compliance of the respiratory system in COVID-19 related ARDS: an international multicenter study
Source: Crit Care. 2021 Feb 8;25:52. doi: 10.1186/s13054-020-03433-0 (PMC7868865; doi:10.1186/s13054-020-03433-0)

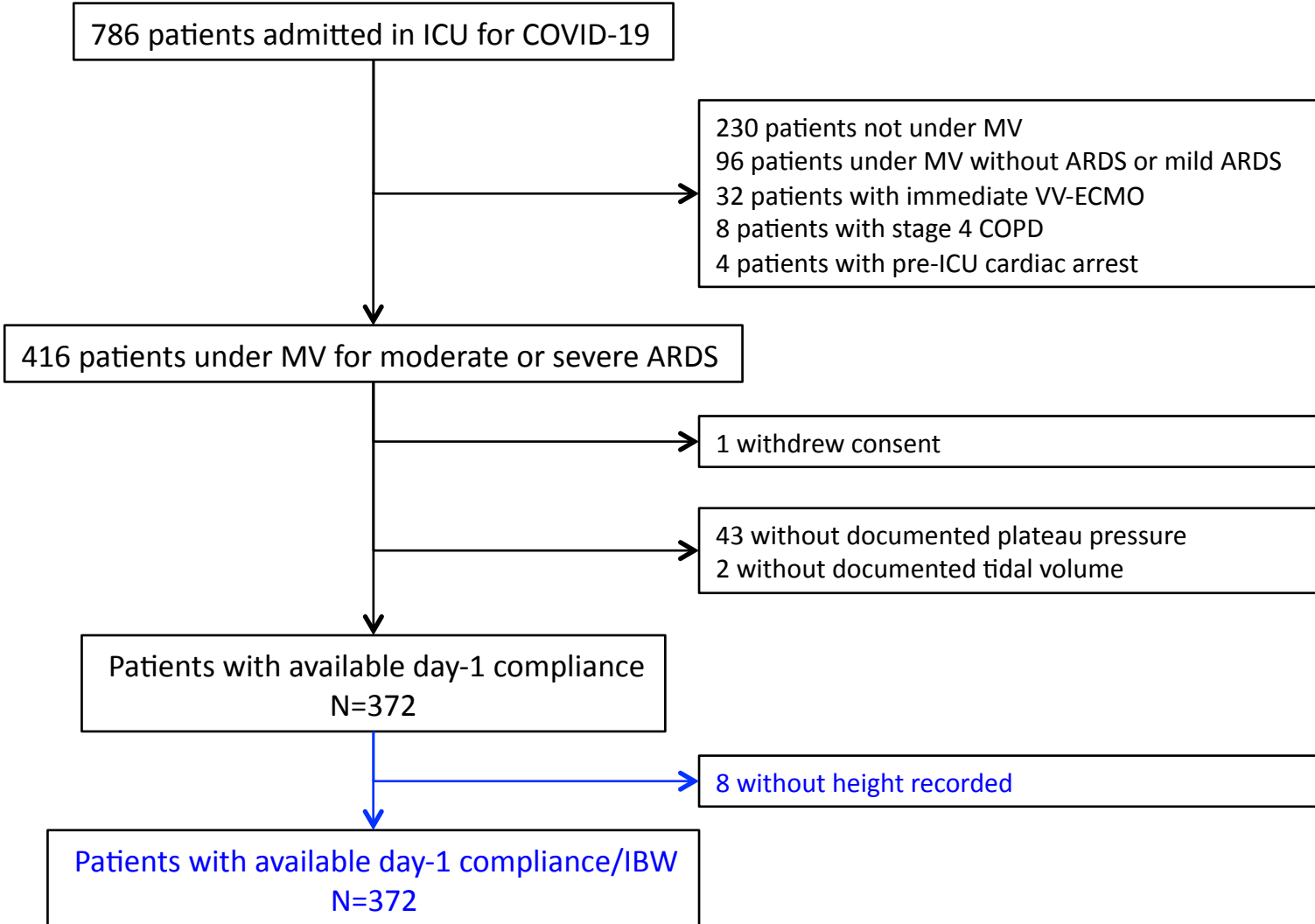

MV: Mechanical ventilation; ARDS: Acute respiratory distress syndrome; IBW: ideal body weight

Supplement: Supplementary file 1 — Additional file 1: Flow chart of the study. [file 13054_2020_3433_MOESM1_ESM.pdf]
