# Supplementary material for: Static compliance of the respiratory system in COVID-19 related ARDS: an international multicenter study
Source: Crit Care. 2021 Feb 8;25:52. doi: 10.1186/s13054-020-03433-0 (PMC7868865; doi:10.1186/s13054-020-03433-0)
Supplement: Supplementary file 2 — Additional file 2: Determinants of day-1 Compliance and Compliance/IBW. [file 13054_2020_3433_MOESM2_ESM.docx]

Online supp Table 1: Determinants of day-1 Compliance and Compliance/IBW

|  | Compliance of the respiratory system (mL/cmH2O) | | | Crs indexed by the IBW (mL/cmH2O/kg IBW) | | |
| --- | --- | --- | --- | --- | --- | --- |
|  | Crude Estimates (CI 95%) ^a^ | *P value*^a^ | adjusted Estimates CI95% ^a^ | Crude Estimates (CI 95%) ^a^ | *P value*^a^ | adjusted Estimates CI95% ^a^ |
| Age, per year | -0.01  (-0.14 ; +0.12) | 0.88 |  | 0.002  (0 ; 0.004) | 0.07 | ns |
| Gender, men | +4.8  (1.7 ; 8.0) | 0.003 | +7.6  (4.4 ; 10.8) | - 0.02  (-0.07 ; + 0.02) | 0.31 |  |
| Body mass index, per kg/m^2^ | -0.1  (-0.4 ; +0.2) | 0.39 |  | -0.001  (-0.005 ; 0.003) | 0.72 |  |
| Chronic Hypertension | -3.2  (-5.9 ; -0.5) | 0.02 | -3.6  (-6.1 ; -1.0) | -0.05  (-0.09-0.006) | 0.02 | -0.05  (-0.08 ; -0.01) |
| Pulmonary chronic disease | -0.2  (-4.2 ; +3.7) | 0.92 |  | 0.005  (-0.05 ; +0.06) | 0.85 |  |
| Charlson comorbidity index | -0.7  (-1.4 ; +0.02) | 0.06 | ns | -0.006  (-0.02 ; 0.004) | 0.25 |  |
| Time from symptoms onset, per days | +0.2  (-0.09 ; 0.6) | 0.16 |  | 0.001  (-0.003 ; 0.005) | 0.67 |  |
| Co-infection | +0.5  (-3.8 ; +4.7) | 0.83 |  | 0.003  (-0.05 ; 0.06) | 0.91 |  |
| Anti-viral treatment^b^ | +3.3  (-0.1 ; +6.7) | 0.06 | ns | 0 (-0.06 ; +0.06) | 0.99 |  |
| PaO2/FiO2 per mmHg | +0.03  (0.003 ; 0.06) | 0.03 | ns | 0.0005  (0.0001 ; 0.001) | 0.02 | 0.0005  (0.0001 ; 0.001) |
| Tidal volume per ml/kg IBW | +2.5  (1.0 ; 4.0) | 0.001 | +4.1  (2.6 ; 5.6) | 0.06  (0.04 ; 0.09) | <0.001 | 0.08  (0.06 ; 0.1) |
| PEEP per cmH_2_O | +1.0  (0.5 – 1.4) | <0.001 | +1 .1  (0.7 ; 1.6) | 0.01  (0.005 ; 0.02) | 0.001 | 0.01  (0.008 ; 0.02) |

IBW: ideal body weight

^a^ Crude and adjusted Estimates and their Confidence Interval (CI) were determined using univariate followed by multivariate linear regression with backward selection. Variables with empty cells were not entered in the model, variables noted “non significant” were eliminate by the backward selection. N=365 patients for the multivariate model on Crs and 361 for the Crs/IBW model

^b^ among lopinavir/ritonavir, hydroxychloroquine, remdesivir
