# Supplementary material for: Static compliance of the respiratory system in COVID-19 related ARDS: an international multicenter study
Source: Crit Care. 2021 Feb 8;25:52. doi: 10.1186/s13054-020-03433-0 (PMC7868865; doi:10.1186/s13054-020-03433-0)
Supplement: Supplementary file 3 — Additional file 3: Crs, Crs/IBW and P/F ratio according to Peep level. [file 13054_2020_3433_MOESM3_ESM.docx]

**online supp Table 2: Crs, Crs/IBW and P/F ratio according to Peep level**

|  | **PEEP 5-8 cmH2O**  **N= 54** | **PEEP 9-12 cmH2O**  **N= 190** | **PEEP > 12 cmH2O**  **N= 120** | **P value ^a^** |
| --- | --- | --- | --- | --- |
| P/F ratio (mmHg), mean (DS) | 121 (±52) | 130 (±49) | 126 (±51) | 0.44 |
| Crs (mL/cmH2O), mean (DS) | 33.8 (±14) | 37.6 (±13) | 39.4 (±14) | 0.04 |
| Crs/IBW (mL/cmH2O/kg), mean (DS) | 0.52 (±0.21) | 0.57 (±0.18) | 0.58 (±0.18) | 0.08 |
| Correlation coefficient between Crs and P/F ^b^ | 0.21 (p=0.13) | 0.11 (p=0.12) | 0.06 (p=0.54) | / |
| Correlation coefficient between Crs/IBW and P/F ^b^ | 0.27 (p=0.045) | 0.12 (p=0.11) | 0.12 (p=0.18) | / |

1. P value was obtained using ANOVA test to compare the 3 groups of patients according to PEEP level
2. The correlation coefficient (R) was calculated using Pearson formula. P value indicates

if the correlation is statistically significant

Crs: Compliance of the respiratory system, IBW: ideal body weight, PEEP: positive end expiratory pressure
